# A reporting checklist for HealthMeasures’ patient-reported outcomes: ASCQ-Me, Neuro-QoL, NIH Toolbox, and PROMIS

**DOI:** 10.1186/s41687-020-0176-4

**Published:** 2020-03-26

**Authors:** Janel Hanmer, Roxanne E. Jensen, Nan Rothrock, on behalf of the HealthMeasures Team

**Affiliations:** 1 Department of Medicine, University of Pittsburgh, 230 McKee Place Suite 600, Pittsburgh, PA 15213 USA; 2 Outcomes Research Branch, Healthcare Delivery Research Program, Division of Cancer Control and Population Sciences, National Cancer Institute, 609 Medical Center Dr, Rockville, MD 20850 USA; 3 Department of Medical Social Sciences, Feinberg School of Medicine, Northwestern University, 625 N. Michigan Ave, Suite 2100, Chicago, IL 60611 USA

**Keywords:** PROMIS, Neuro-QoL, ASCQ-Me, NIH Toolbox, Patient-reported outcomes

## Abstract

**Background:**

ASCQ-Me®, Neuro-QoL™, NIH Toolbox®, and PROMIS®, which are health-related quality of life measures collectively known as HealthMeasures, have experienced rapid uptake in the scientific community with over 1700 peer-reviewed publications through 2018. Because of their proliferation across multiple research disciplines, there has been significant heterogeneity in the description and reporting of these measures. Here, we provide a publication checklist to promote standardization and comparability across different reports. This checklist can be used across all HealthMeasures systems.

Checklist Development: Authors drafted a draft checklist, circulated among the HealthMeasures Steering Committee and PROMIS Health Organization until the members reached consensus.

Checklist: The final checklist has 21 entries in 4 categories: measure details, administration, scoring, and reporting. Most entries (11) specify necessary measure-specific details including version number and administration language(s). Administration (4 entries) reminds authors to include details such as use of proxy respondents and the assessment platform. Scoring (3 entries) is necessary to ensure replication and cross-study comparisons. Reporting (3 entries) reminds authors to always report scores on the T-score metric.

**Conclusion:**

Consistent documentation is necessary to ensure transparent and reproducible methods and support the accumulation of evidence across studies. This checklist promotes standardization and completeness in documentation for ASCQ-Me, Neuro-QoL, PROMIS, and NIH Toolbox measures.

## Background

HealthMeasures (www.healthmeaures.net) is a measurement resource developed by the National Institutes of Health (NIH) to curate, disseminate, and sustain four NIH health-related quality of life focused measurement systems for use in the clinical research community [1]. These systems are: the Adult Sickle Cell Quality of Life Measurement Information System® (ASCQ-Me®) [2], Quality of Life in Neurological Disorders™ (Neuro-QoL™) [3], NIH Toolbox for the Assessment of Neurological and Behavioral Function® (NIH Toolbox®) [4], and the Patient-Reported Outcomes Measurement Information System® (PROMIS®) [5]. Each system within HealthMeasures provides complementary state-of-science approaches to measurement of Patient-Reported Outcomes (PRO). PROMIS is a set of self- and proxy-report measures that assess physical, mental, and social health, symptoms, well-being and life satisfaction in adults and children. PROMIS measures are general, not disease-specific, and are therefore universally applicable within and across disease populations. Neuro-Qol is a measurement system of physical, mental, and social effects experienced by adults and children living with neurological conditions. ASCQ-Me provides measures for adults with sickle cell disease. NIH Toolbox is a comprehensive set of neuro-behavioral measurements that assess cognitive, emotional, sensory, and motor functions. This checklist is only appropriate for the subset of self-report NIH Toolbox measures which were constructed using item response theory; currently these are the Emotion measures which are self- and proxy reports for adults and children. NIH Toolbox measures of cognition, sensation, and motor function are performance-based tests of function and as such, have different types of scores and reporting requirements. More information regarding the reporting of these tests can be found at www.healthmeasures.net/NIHToolbox.

All of these measurement systems have been developed using item response theory (IRT), a modern measurement theory allowing for a wide range of administration and tailoring options [6]. An IRT-calibrated item bank consists of items, each reflecting a level of symptom severity (e.g., anxiety) or function (e.g., physical function). Any number and combination of items from the same bank can be scored and compared to all other measures derived from the same item bank. Item banks enable a wide range of administered forms, from fixed-length paper versions to an electronic, computer adaptive test (CAT). IRT-calibrated fixed-length short forms can be scored using either “response pattern scoring” or with a “look-up” table that converts raw-score totals to T-scores. These options present new requirements for accurately reporting methods and results.

Since becoming available for public use in 2007, measures within the HealthMeasures resource have been adopted in many research applications. On average, nearly 4000 respondent-ready measure PDFs are downloaded each week from HealthMeasures.net. Over 1700 manuscripts related to HealthMeasures were indexed in PubMed between 2004 and 2018. However, as use of these tools increase, there has been considerable variation in information about each measure within publications. Incomplete or incorrect documentation of measures reduces reproducibility and creates challenges for comparisons across studies, systematic review, and meta-analyses. To guide researchers and editorial review, we have developed a checklist to standardize accurate, reproducible documentation of ASCQ-Me, Neuro-QoL, NIH Toolbox Emotion, and PROMIS measures. This checklist is intended as a supplement to other PRO reporting checklists such as CONSORT PRO (Consolidated Standards of Reporting Patient-Reported Outcomes) and COSMIN (COnsensus-based Standards for the selection of health Measurement INstruments) which are focused on study information and systematic reviews rather than measure information [7, 8]. While the checklist presented here was developed for measurement systems within HealthMeasures, it would be appropriate to use this checklist for any measure constructed with IRT techniques.

This checklist is for authors, reviewers, and editors to ensure clear communication of data collection and scoring practices and to improve our ability to interpret results and compare results across publications.

### Checklist development

The authors generated a checklist draft based on information from the HeathMeasures website for each measurement system and their experience reviewing manuscripts for publication. This draft was circulated for feedback by the HealthMeasures Steering Committee and the PROMIS Health Organization Standards Committee until reaching consensus. Participants included measure developers, measurement scientists, psychometricians, and health outcomes researchers from academic and government settings representing all 4 measurement systems. The checklist went through 3 rounds of comments and revisions. Comments that were not included in the checklist were incorporated into the discussion section. After the 3 rounds, all outstanding disagreements were related to terminology rather than content (e.g., is “T-score” capitalized and/or hyphenated?). We present the final checklist in Table 1. The checklist content falls into 4 categories: (1) measure details, (2) administration, (3) scoring, and (4) reporting.

**Table 1:** Reporting checklist for ASCQ-Me, Neuro-QoL, NIH Toolbox Emotion, and PROMIS

Measure Details		
	Requirement	Examples
▢Done	The first mention of a measurement system should include the full name of the measurement system	Adult Sickle Cell Quality of Life Measurement Information System (ASCQ-Me), Quality of Life in Neurological Disorders (Neuro-QoL), NIH Toolbox for the Assessment of Neurological and Behavioral Function(NIH Toolbox), Patient-Reported Outcomes Measurement Information System (PROMIS)
▢Done	Full domain name	Ability to Participate in Social Roles and Activities
▢Done	Version number	v1.2
▢Done	Measure language(s) used in the study	English and Spanish
▢Done	Respondents for whom measure was developed	Adult Pediatric Parent Proxy
▢Done	Measure type	Short form Computer adaptive test (CAT) Profile Scale Uncalibrated item pool Numeric rating scale Checklist
▢Done	Measure citation	Irwin, D., Gross, H., Stucky, B., Thissen, D., Morgan DeWitt, E., Lai, J.S., et al. (2012). Development of six PROMIS pediatrics proxy-report item banks. *Health and Quality of Life Outcomes*, 10 (1), 22.
▢Done ▢N/A	Any modifications to the time frame, item text, or response options.	Because the Time 2 assessment occurred 5 days after Time 1, item text was changed from “In the past 7 days” to “In the past 3 days.”
▢Done ▢N/A	For PROMIS and NIH Toolbox fixed-length short forms: the number of items and any letter designation	Depression 4a PROMIS-29 Profile 5-item Hostility Fixed Form
▢Done ▢N/A	For custom short forms: how the items were selected, the item bank from which items were selected, and the number of items used.	Eight items reflecting poor functioning were selected from the Neuro-QoL v1.0 Lower Extremity Function Mobility Item Bank. Items were selected based upon their item characteristic curves and content.
▢Done ▢N/A	If items or a full measure are reprinted in the publication, permission is required. Contact help@HealthMeasures.net to request permission.	
Administration		
	Requirement	Examples
▢Done	Respondents who completed the measure	Self-report Adult caregivers served as proxy respondents for patients
▢Done	Mode of administration	Unassisted in a private area Mailed to home Interviewer administered in person Interviewer administered by phone
▢Done	Data collection tool	Paper REDCap OBERD NIH Toolbox iPad app Epic PROMIS app
▢Done ▢N/A	Stopping parameters for CAT administration	Standard settings Maximum number of administered items in the CAT was changed from the default to 7
Scoring		
	Requirement	Examples
▢Done	Scoring method	Response-pattern scoring (used by HealthMeasures Scoring Service, REDCap auto-score, Assessment Center API, PROMIS and NIH Toolbox iPad apps)
		Raw sum score to T-score look-up table (from Scoring Manual)
▢Done ▢N/A	Calibration sample used for scoring in the HealthMeasures Scoring Service or Assessment Center API; almost always “default”	Default calibrations
▢Done ▢N/A	Look-up table used for scoring fixed length short forms, scales, and profiles, and discussion of how missing data were handled	Scoring tables published in the PROMIS Adult Profile Scoring Manual. Only short forms with responses to all four items were scored.
Reporting		
	Requirement	Examples
▢Done ▢N/A	Measure scores should be presented as T-scores.	Participants in the intervention group had anxiety symptoms in the moderate range (Neuro-QoL T = 65, SD = 9.0).
▢Done	Keep T-scores on their original scale. Do not reverse scores numerically (e.g., do not change T = 60 to T = 40).	Participants reported moderate impairment in fatigue (T = 62.2, SD = 8.0) and physical function (T = 37.5, SD = 8.1)
▢Done	Report standard deviations with group means	Mean fatigue scores improved from baseline (T = 64.6, SD = 10.0) to follow-up (T = 55.1, SD = 9.5)

## Discussion

The measures within the HealthMeasures family allow an unprecedented amount of flexibility in PRO collection; they also require an unprecedented about of detail in reports of their use. It is important for authors to include these details to improve comparability and reproducibility across different reports. In fact, this reporting checklist is appropriate for use with any PRO measure developed using IRT methods to improve clarity and reproducibility. Our HealthMeasures reporting checklist enumerates the necessary reporting details with 21 entries in 4 topic areas: measure details, administration, scoring, and reporting.

Communicating specific information about the measure in the methods section helps to ensure appropriate transparency and reproducibility. The full measure name, version number, language(s), respondents for whom measure was developed, and measure type should be included at least once but can then be referred to with a shorter label. For example:

PROMIS Parent Proxy Short Form v2.0 - Fatigue 10a in English or Spanish (“Fatigue SF”)

When a report does not include all this information there may be challenges when readers interpret the results. For example, the PROMIS Physical Function v1.0 item bank did not have sufficient discrimination of high functioning individuals where PROMIS Physical Function v2.0 item bank includes more items about higher functioning (e.g., “Are you able to complete 5 push-ups without stopping?”). Therefore, if the PROMIS physical function version is not reported, readers are limited in their evaluation of the measure’s reliability.

In some data collection systems, measures are labeled as “item banks” but are administered as CATs (e.g., REDCap). If the item bank was administered as a computer adaptive test, please refer to the measure as a CAT. For CAT administration, it is also important to note any modifications to the default stopping parameters. Stopping parameters include the maximum number of items administered and error and are listed on HealthMeasures.net. For example, at the time of publication, the PROMIS Adult CAT stopping rules were a minimum number of items of 4 and a stopping rule if the number of items reach 12 or the standard error is below threshold (0.3 on the theta metric or 3.0 on the T-score metric). “Standard settings” is appropriate if the parameters were not changed.

A published validation study of the measure should be referenced and it is appropriate to include both a general validation study and a populations-specific study when available. A list of recommended PROMIS and NIH Toolbox measure citations by domain can be found on HealthMeasures.net (see the “Presenting Results” subsections). If the study used a fixed-length short form without its own publication, citing the publication describing the development of the full item bank is appropriate. There is a list of PROMIS, Neuro-QoL, ASCQ-Me, and NIH Toolbox publications within each of their respective sections of the website (see the “Publications” subsections).

Reporting how a measure was administered is also important for interpretation of the study results. Each measure was developed and tested for a specific respondent (e.g., adult and pediatric self-report, pediatric parent-proxy report). Explicit documentation of modifications enables evaluation of the validity of the collected data. However, there are settings and situations in which deviations from these administration methods are needed. We also encourage authors to report any evaluation of the modifications that they performed to extend our collective knowledge about measure performance and facilitate continued measure improvement. However, if a modification is to be adopted by HealthMeasures, it needs to be submitted for review at HealthMeasures.

Scoring is the smallest checklist category but may be the most critical for cross-study comparisons and will facilitate any future data pooling efforts. Each measure’s score is designed to be reported on a T-score metric that has a mean of 50 in an established reference general population allowing for normative interpretation of scores. Guidance for the interpretation of scores is available on the HealthMeasures website in the “Score and Interpret” section.

Raw scores and/or percentiles may be reported in addition to, but not in place of, T-scores. If only raw scores are reported, analyses on these scores are inaccurate and lose the many advantages of IRT-based measures (e.g., measurement precision, score interpretability). It should be noted that a very small number of PROMIS, Neuro-QoL, and ASCQ-Me measures do not produce IRT-based T-scores (e.g., item pools, single item numeric rating scales, checklists). In these cases, the raw score alone can be reported. More information is included in each measure’s scoring manual. Additionally, although look-up tables are acceptable for scoring standardized short forms, missing data dramatically alter their precision of the estimated T-score. It is therefore important to describe scoring procedures and the management of missing data.

T-scores should never be “reverse scored” (e.g., do not change T = 60 to T = 40). Neuro-QoL, NIH Toolbox Emotion, and PROMIS T-scores are designed such that higher T-scores indicate more of the concept being measured. For measures of function, a higher score is better health (e.g., physical function); for measures of symptoms, a higher score is worse health (e.g., fatigue). When a study includes both function and symptoms measures, authors should be aware of the potential for confusion in their readers. Some authors have been tempted to numerically reverse some of the scores (e.g., higher numbers always indicate worse health). However, if authors make this score modification, they increase the likelihood of misinterpretation both by readers familiar with the measures and when studies are compared.

**Fig. 1:**
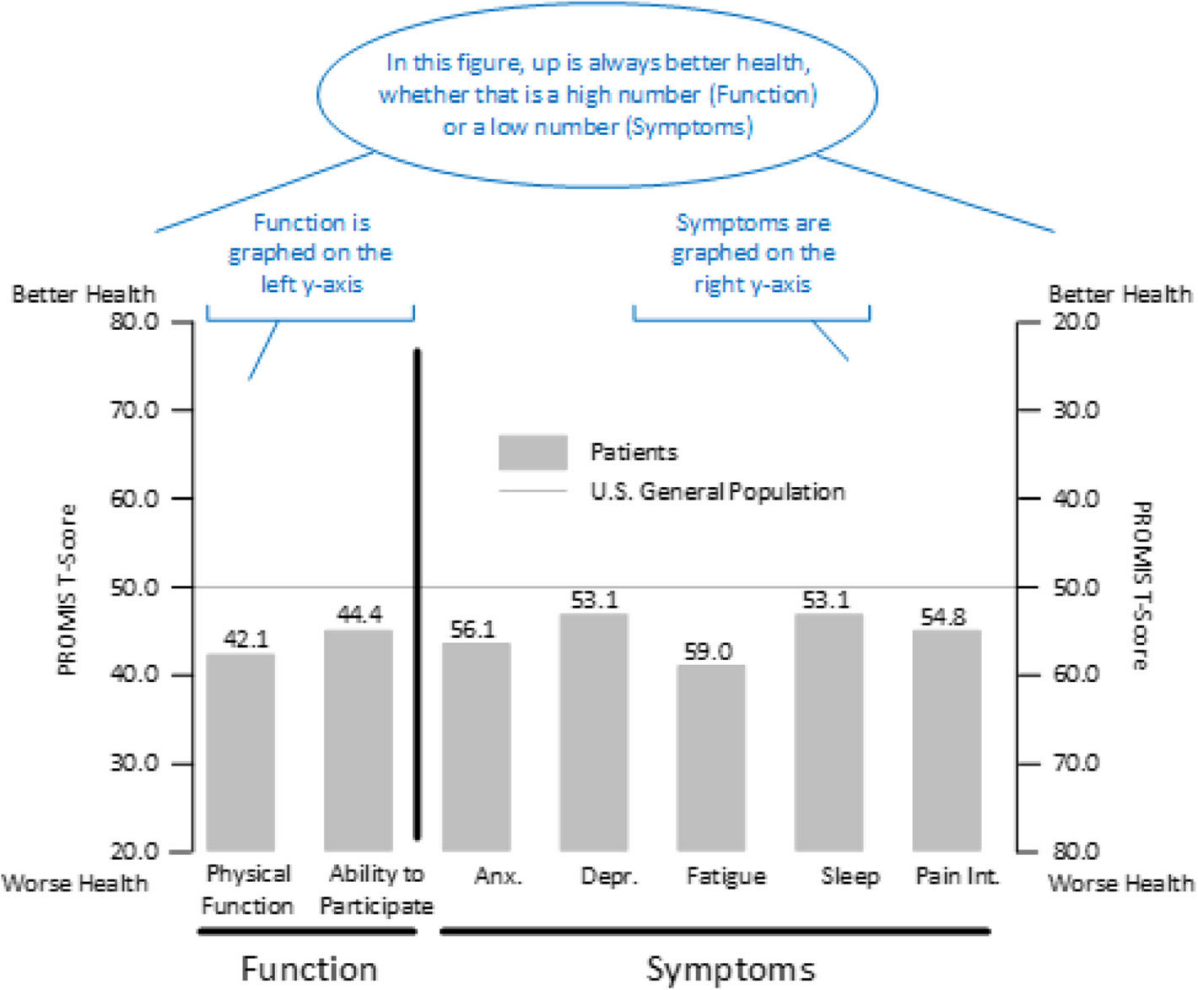
Example results from the PROMIS-29 Profile v2.1. Notes are in blue

**Fig. 2:**
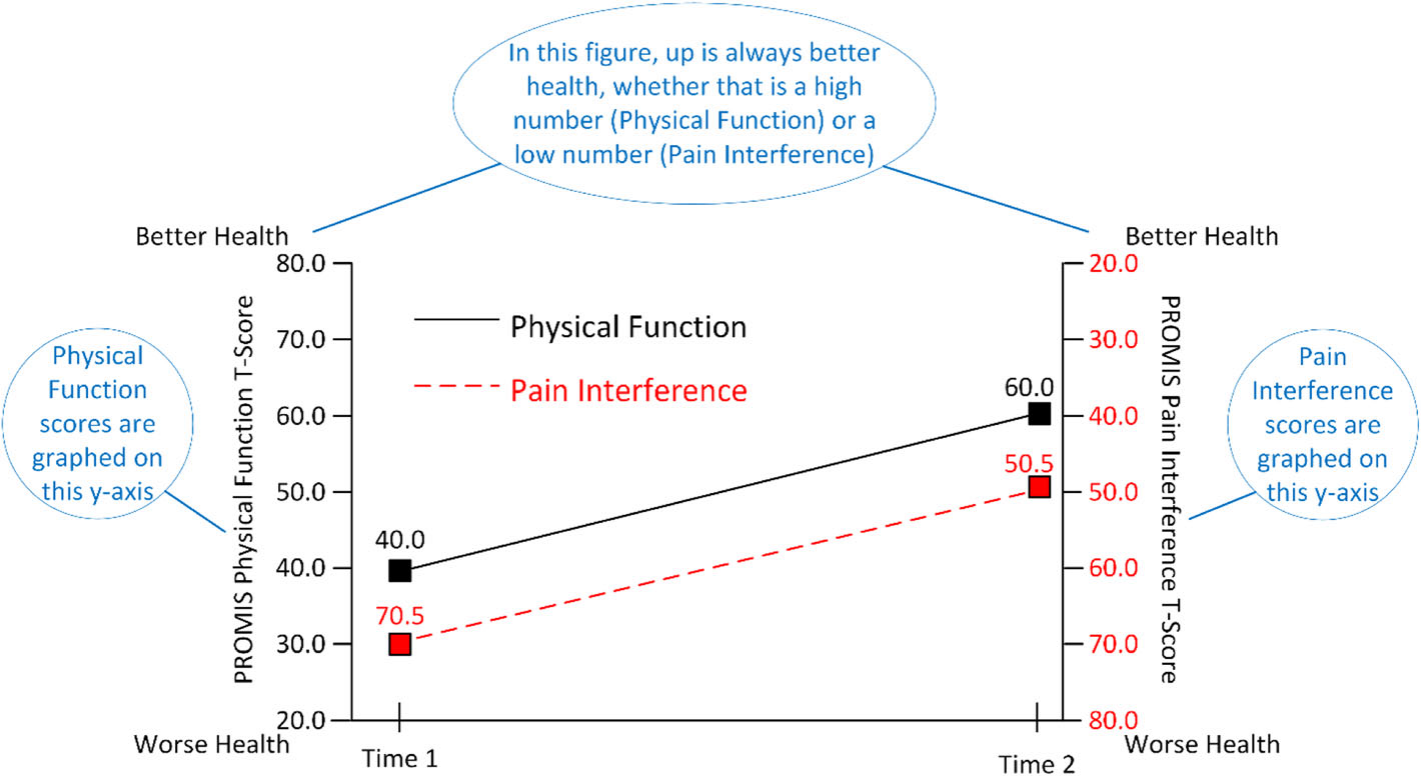
Example results of change in function and symptom scores over time. Notes are in blue

If a study includes both function and symptom measures, it is easiest for readers if the symptom measures are grouped together and the function measures are grouped together. Ideally, an author would have the space to present the results in different figures or as different panels within a figure. However, sometimes authors must combine symptom and function measures into a single figure because of publisher requirements. If a combined figure is required, we recommend utilizing graphs with two y-axes. Figures 1 and 2 provide examples of this approach.

Figure 1 shows a hypothetical group of patients reporting worse health than the U.S. General Population in all domains. Function measures are grouped together and use the left-side x-axis. Symptom measures are grouped together and use the right-side x-axis. Figure 2 displays longitudinal data with “up” indicating better health. Patients are improving in both Physical Function (left y-axis) and Pain (right y-axis). Note that the y-axis for these figures ranges from 20 to 80 which is generally sufficient for T-scores as it displays the mean +/− 3 standard deviations. Use of 0 and 100 as anchors can be misinterpreted as percentiles and visually reduce magnitude.

Here is an example of methods and results that fulfill the checklist:

Methods: The Quality of Life in Neurological Disorders (Neuro-QoL) Lower Extremity Function – Mobility CAT v1.0 (Mobility CAT) for adults (Gershon et al 2012) was administered in English. Participants completed the measures unassisted in REDCap on a tablet computer in a private area. REDCap uses standard CAT settings and response-pattern scoring with default calibrations.

Results: At baseline, participants reported some mobility impairment (Mobility CAT mean T-score=38.0, SD=7.6)*.*

## Conclusions

ASCQ-Me, Neuro-QoL, NIH Toolbox Emotion, and PROMIS are increasingly popular measures of health-related quality of life. To ensure consistency and completeness in reporting these measures, we used the combined expertise of the HealthMeasures Steering Committee and PROMIS Health Organization to make an explicit reporting checklist. This checklist is appropriate for use with any PRO measure developed using IRT methods. Use of this checklist will improve the quality of publications, accuracy of the interpretation of results, and improve cross-study comparisons. A pdf of the checklist is in an online supplement (Additional file 1) and on the HealthMeasures website (www.HealthMeasures.net).

## Supplementary information

**Additional file 1.** Checklist.

## Data Availability

Not applicable.

## Abbreviations

ASCQ-Me®: Adult Sickle Cell Quality of Life Measurement Information System®
CAT: Computer Adaptive Test
CONSORT PRO: Consolidated Standards of Reporting Patient-Reported Outcomes
COSMIN: COnsensus-based Standards for the selection of health Measurement INstruments
IRT: Item Response Theory
Neuro-QoL™: Quality of Life in Neurological Disorders™
NIH Toolbox®: NIH Toolbox for the Assessment of Neurological and Behavioral Function®
NIH: National Institutes of Health
PRO: Patient-Reported Outcomes
PROMIS®: Patient-Reported Outcomes Measurement Information System®
