# Supplementary material for: A reporting checklist for HealthMeasures’ patient-reported outcomes: ASCQ-Me, Neuro-QoL, NIH Toolbox, and PROMIS
Source: J Patient Rep Outcomes. 2020 Mar 26;4:21. doi: 10.1186/s41687-020-0176-4 (PMC7096598; doi:10.1186/s41687-020-0176-4)
Supplement: Supplementary file 1 — Additional file 1. Checklist. [file 41687_2020_176_MOESM1_ESM.pdf]

Status	Requirement	Examples
**Measure Details**		
☐Done	The first mention of a measurement system should include the full name of the measurement system	Adult Sickle Cell Quality of Life Measurement Information System (ASCQ-Me); Quality of Life in Neurological Disorders (Neuro-QoL); NIH Toolbox for the Assessment of Neurological and Behavioral Function(NIH Toolbox); Patient-Reported Outcomes Measurement Information System (PROMIS)
☐Done	Full domain name	Ability to Participate in Social Roles and Activities
☐Done	Version number	v1.2
☐Done	Language(s) used in the study	English and Spanish
☐Done	Respondents for whom measure was developed	Adult; Pediatric; Parent Proxy
☐Done	Measure type	Short form; Computer adaptive test (CAT); Profile Scale; Uncalibrated item pool; Numeric rating scale; Checklist
☐Done	Measure citation	Irwin, D., Gross, H., Stucky, B., et al. (2012). Development of six PROMIS pediatrics proxy-report item banks. *Health and Quality of Life Outcomes*, 10(1), 22.
☐Done<br>☐N/A	Any modifications to the time frame, item text, or response options.	Because the Time 2 assessment occurred 5 days after Time 1, item text was changed from "In the past 7 days" to "In the past 3 days."
☐Done<br>☐N/A	For PROMIS and NIH Toolbox fixed-length short forms: the number of items and any letter designation	Depression 4a; PROMIS-29 Profile; 5-item Hostility Fixed Form
☐Done<br>☐N/A	For custom short forms: how the items were selected, the item bank from which items were selected, and the number of items used.	Eight items reflecting poor functioning were selected from the Neuro-QoL v1.0 Lower Extremity Function Mobility Item Bank. Items were selected based upon their item characteristic curves and content.
☐Done<br>☐N/A	If items or a full measure are reprinted in the publication, permission is required. Contact help@HealthMeasures.net to request permission.	
**Administration**		
☐Done	Respondents who completed the measure	Self-report; Adult caregivers served as proxy respondents for patients
☐Done	Mode of administration	Unassisted in a private area; Mailed to home; Interviewer administered in person; Interviewer administered by phone
☐Done	Data collection tool	paper; REDCap; OBERD; NIH Toolbox iPad app; Epic PROMIS app
☐Done<br>☐N/A	Stopping parameters for CAT administration	standard settings, maximum number of administered items in the CAT was changed from the default to 7
**Scoring**		
☐Done	Scoring method	response-pattern scoring (used by HealthMeasures Scoring Service, REDCap auto-score, Assessment Center API, PROMIS and NIH Toolbox iPad apps); raw sum score to T-score look-up table (from Scoring Manual)
☐Done<br>☐N/A	Calibration sample used for scoring in the HealthMeasures Scoring Service or Assessment Center API; almost always "default"	default calibrations
☐Done<br>☐N/A	Look-up table used for scoring fixed length short forms, scales, and profiles, and discussion of how missing data were handled	Scoring tables published in the PROMIS Adult Profile Scoring Manual. Only short forms with responses to all four items were scored.
**Reporting**		
☐Done<br>☐N/A	Measure scores should be presented as T-scores.	Participants in the intervention group had moderate anxiety symptoms (Neuro-QoL T=65, SD=2.0).
☐Done	Keep T-scores on their original scale. Do not reverse scores numerically (e.g., do not change T=60 to T=40).	Participants reported moderate impairment in fatigue (T=62.2, SD=2.0) and physical function (T=37.5, SD=2.1)
☐Done	Report standard deviations with group means	Mean fatigue scores improved from baseline (T=64.6, SD=2.0) to follow-up (T=55.1, SD=2.5)
